# Utility of Telehealth Platforms Applied to Burns Management: A Systematic Review

**DOI:** 10.3390/ijerph20043161

**Published:** 2023-02-10

**Authors:** Antonio García-Díaz, Lluís Vilardell-Roig, David Novillo-Ortiz, Purificación Gacto-Sánchez, José Juan Pereyra-Rodríguez, Francesc Saigí-Rubió

**Affiliations:** 1 Plastic Surgery and Major Burns Service, Virgen del Rocío University Hospital, 41013 Seville, Spain; 2 Faculty of Health Sciences, Universitat Oberta de Catalunya (UOC), 08018 Barcelona, Spain; 3 Division of Country Health Policies and Systems, Regional Office for Europe, World Health Organization, 2100 Copenhagen, Denmark; 4 Dermatology Service, Virgen del Rocío University Hospital, 41013 Seville, Spain

**Keywords:** telehealth, remote consultation, burns, cost–benefit analysis

## Abstract

The financial burden of burn injuries has a considerable impact on patients and healthcare systems. Information and Communication Technologies (ICTs) have demonstrated their utility in the improvement of clinical practice and healthcare systems. Because referral centres for burn injuries cover large geographic areas, many specialists must find new strategies, including telehealth tools for patient evaluation, teleconsultation, and remote monitoring. This systematic review was performed according to PRISMA guidelines. PubMed, Cochrane, Medline, IBECS, and LILACS were the search engines used. Systematic reviews, meta-analyses, clinical trials, and observational studies were included in the study search. The protocol was registered in PROSPERO with the number CRD42022361137. In total, 37 of 185 studies queried for this study were eligible for the systematic review. Thirty studies were comparative observational studies, six were systematic reviews, and one was a randomised clinical trial. Studies suggest that telehealth allows better perception of triage, more accurate estimation of the TBSA, and resuscitation measures in the management of acute burns. In addition, some studies assess that TH tools are equivalent to face-to-face outpatient visits and cost-efficient because of transport savings and unnecessary referrals. However, more studies are required to provide significant evidence. However, the implementation of telehealth should be specifically adapted to each territory.

## 1. Introduction

Information and Communication Technologies (ICTs) have demonstrated their utility in clinical practice and in the improvement of healthcare systems by finding practical solutions to routine problems. Telehealth (TH), which emerged within this context, is defined as the use of ICTs to enable the transfer of medical information for diagnostic, therapeutic, and educational purposes, regardless of physical location [1]. TH has earned its place in the assessment and evaluation of burn patients (Teleburn), and the importance of this tool has become evident [2]. Although the evaluation of the extent and depth of burns requires significant experience, the use of TH for non-specialist professionals could be adequately managed [3,4,5,6,7]. In addition, the treatment of burns requires a high degree of specialisation, which is concentrated in tertiary hospitals that often cover large geographic areas. Thus, TH technologies have considerable potential in the proper triage of serious burns that, as true medical emergencies, need early treatment [8]. TH allows burns images to be sent immediately, identifying those patients who should be urgently transferred to specialist centres [9,10]. 

The application of TH in burn care fosters close collaboration between experts and other healthcare professionals, creating a patient-centred environment and an optimised multidisciplinary framework [11]. Likewise, it strengthens care networks, particularly for populations with access barriers and for regions in which certain medical specialties are underdeveloped, such as rural communities [12]. 

Adequate and efficient use of TH for burn patients requires coordination among professionals and investment in certain areas. This study aims to critically evaluate the literature on the cost–benefit impact of TH in burn patients and to investigate the clinical effectiveness of implementing TH strategies.

## 2. Materials and Methods

### 2.1. Search Strategy

A systematic review of the available literature was performed, and the quality and homogeneity of the studies were assessed. PubMed, Cochrane, Medline, IBECS, and LILACS were the search engines used. This systematic review was performed according to the Preferred Reporting Items for Systematic Reviews and Meta-Analyses (PRISMA) [13]. Before the study was carried out, the protocol was registered in PROSPERO with the number CRD42022361137. The syntax used for each database was the following:

Medline (accessed via PubMed): (“Telemedicine” [Mesh] OR “Remote Consultation” [Mesh] OR “Telehealthcare” OR “Telemonitoring” OR “remote monitoring” OR “Telediagnosis” OR “tele-management” OR “Teleconsult”) AND (“Cost-Benefit Analysis” [Mesh] OR “Evaluation Studies” [Publication Type] OR “Program Evaluation” [Mesh] OR Impact OR Effectiveness OR Efficacy OR Cost efficiency OR Cost effectiveness OR Benefit OR Efficiency) AND (“Burns” [Mesh] OR “Teleburns” OR “Burns” OR “Burn” OR “Burned” OR “Scorch”).

IBECS, LILACS, Cochrane (accessed via the Virtual Health Library (VHL)): Telemedicine AND ((analysis* AND cost-Benefit) OR cost OR effectiveness OR cost-utility OR (economic AND evaluation) OR (program AND evaluation) OR impact OR effectiveness OR “clinical trial” OR (program AND sustainability)) AND Burns.

### 2.2. Selection Criteria

Peer-reviewed publications categorized as systematic reviews, meta-analyses, and clinical trials assessing the effectiveness and cost-effectiveness of ICT use in the medical care of burn victims were initially included in the study search. Given the very low number of results returned, the inclusion criteria were expanded to include comparative observational studies.

The selection of studies was divided into four phases, as described in the PRISMA framework [13]. The first phase (identification) consisted of collecting all articles retrieved from the databases (174 titles). This first stage of article selection was based on titles and abstracts. Any abstract that did not provide enough information to evaluate the intervention or methodology according to the defined inclusion and exclusion criteria could be recovered in the full-text review stage. This process was performed by two independent reviewers (LVR, JJPR) who examined each article in parallel. An article was considered to have passed to the next stage if at least one reviewer marked it as relevant. After eliminating duplicates, 156 titles remained. During the second stage (screening), the two researchers reviewed all entry titles, and subsequently, they checked the eligibility of all studies (third stage) using inclusion and exclusion criteria. The selected studies needed to be related to the effectiveness or cost–benefit analysis of burn victim interventions using TH, written in English, and published between 1 January 2001 and 30 September 2022. The most important data items extracted from the studies included in the review were the type of study, the characteristics of the interventions aimed at burn victims with relevant details of the intervention delivered using TH, the clinical outcomes of this intervention, and the results of assessing the impact on clinical or financial indicators. The following were specifically excluded: (i) feasibility, user acceptance, and usability studies that did not evaluate the impact on clinical or financial indicators; (ii) studies that only evaluated “perceived benefits;” and (iii) non-systematic reviews and case reports. If initially, the screening was based on titles and abstracts, and articles were independently assessed by both independent reviewers, pre-selected records that were deemed possibly eligible were thoroughly examined based on full-text assessment. Publications could be added using the reference lists of the selected manuscripts. Authorship, journal, or years were not blinded. In case of doubt or disagreement, a third reviewer was involved in the selection decision (FSR), and the disagreement was resolved by consensus discussions.

### 2.3. Data Extraction

After reading the selected articles, some of the following details were collected: type of study, number and characteristics of patients included, and, if possible, an evaluation of the costs. Table 1 was constructed from these data. Data were individually extracted and cross-checked for accuracy by a second researcher. Both researchers (LVR and JJPR) reviewed the table and analysed common patterns, contradictory results, and gaps between studies. All identified items were presented and discussed with the other four investigators (AGD, DNO, PGS, and FSR).

### 2.4. Synthesis

Once the data were qualitatively synthesised, they were critically discussed by the investigators, both qualitatively and quantitatively, based on the following PICOS criteria:

- P (population): patients requiring treatment of their burns with a high degree of specialisation, particularly in tertiary hospitals;
- I (intervention): TH;
- C (comparison): conventional treatment versus treatment using TH;
- O (outcomes): the cost-benefit impact of TH in burn patients, as well as the clinical effectiveness of implementing any TH strategy;
- S (study design): any.

In the case of missing information, we planned to contact the authors of the studies by e-mail. However, this method was not necessary to collect essential data.

The certainty in the body of evidence regarding the efficacy of TH intervention for burn patients was evaluated with the internationally recognized four-tier (A, B, C, D) grade system [14].

## 3. Results

**Figure 1:**
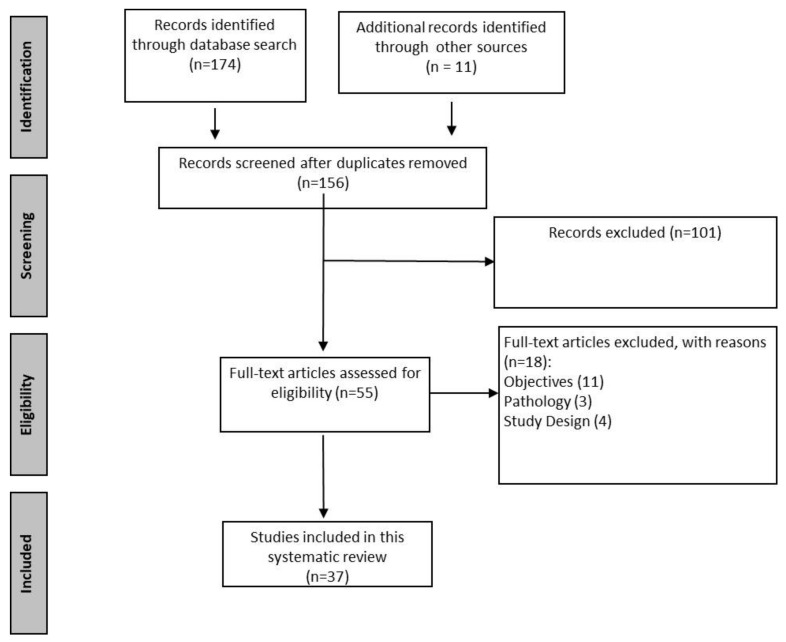
Flow chart summarising the results of the screening process and final article selection.

A total of 37 articles were included. Figure 1 shows the flow chart summarising the screening process. Most of the articles were published in the last decade, mainly from 2016 to date (Figure S1). Of these, the majority were comparative observational studies (13 cohort studies, 10 retrospective studies, 3 case-control studies, and 4 transversal studies) [15] (Table 2). Six systematic reviews and one randomised clinical trial (RCT) were also included. The global evaluation of the evidence according to the grading system was moderate (Table 1).

### 3.1. Clinical Results in the Management of Acute Burns

Of the 37 articles included, 35 referred to the management of acute burns and 17 studies mentioned an improvement and/or better perception of triage. More specifically, 12 studies were closely related to a more accurate estimation of the total body surface area (TBSA) burned [2,12,15,16,21,23,24,25,26,29,43,45], while only 1 stated that the TH was accurate in patients with intermediate-size burns measuring 1–10% TBSA [31]. With greater accuracy due to TH, healthcare staff were better guided in their clinical decisions (eight articles) [2,7,18,20,21,25,41,43], and resuscitation measures—including fluid therapy—were more accurate and effective (four articles) [6,16,18,24].

Most of the studies noted that TH was just as effective as acute bedside management, while three studies showed that TH was even better [7,18,25]. Gacto-Sánchez et al. [28] noted the usefulness of TH for referral planning. Lewis et al. found that TH in the management of acute burns was better than conventional evaluation in rural and resource-poor areas, offering more appropriate and better-quality care. Parviz et al. showed that if TH was not used, deviations in the calculation of TBSA were high across professionals, with an overestimation of up to 230% [25]. Wibbenmeyer et al. mentioned better effectiveness in interprofessional communication in the emergency management of burn victims in rural areas [18].

### 3.2. Clinical Results in the Follow-Up of Burn Patients

Regarding follow-up, monitoring, and rehabilitation of burn patients, 14 articles considered TH as a potentially beneficial tool [2,30,32,33,34,35,36,38,39,40,41,42,43,44].

In eight studies, TH tools, essentially static images and/or photographs via smartphones, and dynamic images through videoconferencing, managed to achieve remote clinical follow-up of a standard equivalent to face-to-face outpatient visits [2,34,36,37,39,42].

### 3.3. Results in Cost Evaluation

Costs were explicitly mentioned in most of the articles. However, some studies did not provide cost analyses, assuming that TH tools were cost-efficient (appropriate triage, transport savings, fewer unnecessary referrals, etc.). In seven articles, the integration of TH, and videoconferencing in particular, was perceived as highly satisfactory by the patients because of time and transport savings [2,15,19,34,36,42]. In contrast, the study by Cai et al. showed that patients had a less favorable opinion of videoconferencing than they did of face-to-face management [12].

Sixteen studies mentioned the patients’ transport savings, and some of them focused on the reduction in air transfers, which were mainly linked to a lower overestimation of the injury (better triage for better TBSA accuracy) [6,15,16,18,20,21,24,27,32,33,39,41,42,43,44,46]. Along similar lines, eleven studies reported savings in terms of referrals for both acute burns and postoperative follow-up [8,16,21,28,33,38,39,41,42,43,46]. In their respective studies, Liu et al. and Hickey et al. mentioned that there had been no readmissions to the burn centre of patients followed up by TH, suggesting that clinical decisions were accurate [32,33]. It should be noted that a high proportion of the articles found that the TH option had been highly beneficial in rural areas.

Four articles [21,32,34,43] quantified the number of inpatient days avoided because of the use of TH, while two studies quantified the number of beds freed up by TH, thus leaving room for patients who needed to be admitted to a burn centre according to the relevant triage [32,44].

In addition, four studies [2,8,27,35] concurred that the implementation and use of TH tools were initially expensive for healthcare providers (a major investment in technology and training). However, most of them found that TH represented a substantial saving for the patient.

Several studies, and two in particular [7,43], reflected the professionals’ lack of education and training when it came to using these new technologies for diagnosing and treating their patients, which was associated with some professionals’ reluctance toward eHealth tools. However, seven articles placed particular emphasis on better interprofessional communication that resulted from the implementation of TH tools [6,18,33,35,38,41,43]. Twenty-one studies found shortcomings relating not only to ethical, legal, and regulatory issues but also to security, confidentiality, and privacy. They also noted that limitations existed regarding interoperability and compatibility between systems using TH [2,7,12,15,16,17,19,21,23,27,28,33,35,36,37,38,40,42,45,46]. Besides these shortcomings, the impact of which means that such technologies run into complex implementation difficulties, there is a need for more studies to investigate how significant improvements could be achieved in the clinical efficacy and cost-efficiency associated with their use.

Different studies provided different evidence in the cost analysis of TH with acute burns. This is the case of Redlick et al. (2002) [36], Nguyen et al. (2004) [35], Saffle et al. (2004) [21], and Smith et al. (2007) [27]. In a retrospective Australian study conducted in 2013, McWilliams et al. identified savings over eight years of 4905 inpatient bed days, 364 acute patient transfers, and 1763 patient follow-up review transfers for a total of 1312 pediatric burn patients because of this teleburn service [44]. This study presented an estimation of savings in the 2012–2013 period of AUD 1.89 million [44].

Russell et al. (2015) mentioned that, over three years, TH consultations at the burn centre had almost tripled, air transport had fallen from 100% to 44% of consults, and burn severity of those patients transported had increased (better triage) [42]. A separate sample of 24% of TH visits between 2010 and 2011 showed that these visits directly resulted in USD 4.2 million in revenue to the University of Utah [42]. This success essentially resulted in expanded institutional efforts in TH. Similar results are presented by Gacto-Sanchez and Garber [28,30]. These studies demonstrated a reduction of transfers by incorporating triage of acute burns by TH. Specifically, Garber estimated that between USD 150,000 and USD 180,000 were saved in air transportation costs in the triage of 155 patients.

In 2017, Liu et al. conducted a retrospective study on 29 patients enrolled in 73 virtual rehabilitation visits carried out via videoconferencing in the period between 2013 and 2014 in the United States. During that period, total transport cost savings of USD 101,110 were achieved by eliminating 146 ambulance transfers [32]. Because of the reduction in time of virtual visits, 6.8 days in outpatient burn management and 80 inpatient bed days were saved [32]. At the same time, the rehabilitation centre reduced interruptions and reported better efficiency for the hospital [32].

In addition, Hickey et al. (2017) reviewed 31 burn patients that had taken part in the follow-up programme via Interactive Home Telehealth (IHT) videoconferencing for 15 months (2015–2016) [33]. The study found that the average roundtrip distance saved was 188 miles and that the patients’ average roundtrip travel time saved was 201 min [33]. Along similar lines, Martínez et al. (2018) estimated that 160 admissions were avoided because of better triage through 838 communications via WhatsApp in both acute management and subsequent follow-up [41].

In their attempt to evaluate the effectiveness and cost savings of virtual home visits (VV) during six months of the COVID-19 pandemic, Head et al. (2022) [45] calculated that VV saved 130 miles per encounter, 164 min in travel time and USD 185 (USD 104 in driving expenses and USD 81 in wages retained). The total amount saved for all visits during the six-month timeframe was 7929 miles, 10,001 travel minutes, USD 6287 driving costs, and USD 4912 wages. The total estimated financial savings of VV was USD 11,199 (distance *p* < 0.001; time *p* < 0.001; driving cost *p* < 0.001; foregone wages *p* < 0.001).

## 4. Discussion

The impact of ICTs is increasing exponentially, especially for mobile technologies. Although their introduction into healthcare systems remains complex, the TH tools are becoming increasingly familiar and accessible. This issue justifies the increase in the number of teleburn-related articles in the past decade. 

Burn patient care is organised around referral centres covering large geographic areas [2]. It has been estimated that burn injuries cause around 265,000 deaths annually worldwide [47]. In addition, immediate care for major burn patients is crucial. Thus, optimal and effective emergency management involving the use of TH early on in the post-injury period may prevent a burn from getting worse, facilitating quicker care and rehabilitation, and consequently, better long-term functionality [6].

On the basis of data from cohort studies, retrospective studies, case-control studies, transversal studies, systematic reviews, and RCT from different European countries (5), Asia (3), Africa (2), the United States, Canada, and Australia, the results showed clinical benefits of TH interventions in the management of acute burns, clinical decisions, triage, follow-up, monitoring, and rehabilitation of burn patients. TH tools are shown to be effective in providing interprofessional access and communication for expert advice, and TH interventions are cost-efficient in appropriate triage of acute burns, transport savings, and reduction in the number of unnecessary referrals, increasing patient satisfaction experience because of time and transport savings. 

The fact that the largest number of experiences analyzed were from the USA (with 18) and Australia (with 5) leads us to believe in the suitability of the service due to the large extension of the territory and the benefits that the TH intervention brings to the patient by saving travel time. The population density in some areas of these countries can be low, which makes it difficult to have a cost-effective health system in these areas. These experiences aim to improve cost-effectiveness through the use of technologies such as TH and thus contribute to reducing the costs of providing health services over long distances. Thus, cost-effectiveness in triage and follow-up in larger countries, such as the USA and Australia, has been more feasible and viable, as costs also have a much greater impact. However, it is important to bear in mind that acute care and follow-up treatment and/or inpatient management are different clinical scenarios that give rise to a variety of study settings. Although older studies (Wallace et al., 2007 [19], 2008 [8]) pointed out the significant capital outlay and no evidence of cost savings, as technology has become more affordable, it has become more accessible to a wider range of clinical scenarios. This can help to expand access to specialized care for burn patients. The advancements in technology, especially the internet and mobile devices, allow for remote monitoring and telemedicine, which can help to improve outcomes for burn patients, by giving them access to specialists, reducing the need for travel, and promoting continuity of care. This can be particularly important for burn patients who require ongoing care and monitoring, as well as for patients with burn-related complications.

Most of the articles focused on analysing TH in the acute burn stage, and the results show that injury overestimation (calculation of TBSA) in triage for transport (often by air because of the long distances mentioned) could have been avoided in many instances by using TH technologies based on image transfer and videoconferencing. However, few studies reported any significant evidence of cost efficiency. The quality of diagnostic management in the majority of cases was nevertheless shown to be equal to (and not lower than) face-to-face care, but there is still a shortage of high-level evidence studies showing that TH-assisted care is better. According to the studies analysed, efforts are being made to strengthen the viability of TH to promote better triage, prevent unnecessary emergency transfers, and provide better support for clinical decisions in the acute resuscitation and subsequent follow-up stages. The use of TH could empower non-specialist professionals to manage minor burns in their centres. Communication via mobile phone has become a reliable method in the assessment of burn injuries using TH [24,29]. In addition, significant aspects found in the clinical results, such as triage accuracy, indirectly reinforce that TH is a cost-efficient tool: time and cost savings result from a reduction in referrals, transport, and unnecessary readmissions (inpatient days, bed occupancy), among others.

Moreover, a small number of the studies focused on chronic follow-up of these patients via virtual visits, testing whether TH was a useful tool for monitoring during burn patient rehabilitation. Remote clinical follow-up of a standard equivalent to outpatient bedside clinical consultation was achieved, the indirect costs of transport fell, hospital resources (unnecessary admissions and clinic appointments) were saved, and patient satisfaction increased (closely linked to the reduction in long trips). In addition, the reduction of travel time improved adherence to the rehabilitation care plan without reducing the quality of care [32].

Although previous studies in TH tried to provide evidence of significant improvements in cost-efficiency, the viability of those is complex for several reasons [47]. According to Hasselberg, the implementation of image-based mHealth systems and the relevant evaluation of their impact on health should be based on face-to-face care (gold standard); however, their design might involve a wide range of ethical and practical issues. For example, the time lapse between patient recruitment and study results might be critical because that technology may have become outdated before a study has been completed [47].

The results from the RCT conducted by Burgess et al. (2018) showed that the use of a mobile app to help parents prevent scalds in children achieved greater overall knowledge scores in parents who had completed their education [17]. However, a change in behaviour could not be extrapolated.

Some studies pointed to barriers that should be considered when planning and implementing TH interventions: Deficiency in technology-related knowledge and skills of health professionals and their reluctance toward TH tools on the one hand; lack of definitive scientific evidence on its clinical contribution, on the other; patients’ feeling of having less personal contact with the clinician during videoconferencing and face-to-face preference on the other; and barriers associated with ethics, security, and privacy issues, along with connectivity, interoperability, and compatibility between systems using TH, on the other, hinder the implementation of these technologies. These barriers and challenges associated with the use of TH are in line with other studies [48,49]. The effectiveness that these TH interventions can deliver in the management of burn patients should facilitate the implementation of these applications, as well as recognise and address the drawbacks to maximise the likelihood of their successful use. Research faces the challenge of producing such evidence, a prerequisite for the widespread adoption of TH in burns management.

### Limitations

A total of five databases were explored, focusing only on systematic reviews, meta-analyses, and clinical trials, thus limiting the exhaustivity of the search. Although we initially identified almost 185 studies for screening, our screening found 37 studies meeting our inclusion and exclusion criteria. The large time span of more than 20 years (between 1 January 2001 and 30 September 2022) may have influenced the effect of technology evolution in the context of cost analysis and effectiveness. The fact that the technology has become cheaper and more readily available may have more effects on the cost analysis than those addressed in our study.

## 5. Conclusions

The most widely used TH tools are videoconferencing and photographs via smartphone. TH interventions allow a better perception of triage, more accurate estimation of the TBSA, and resuscitation measures in the management of acute burns and decision-making. In addition, some studies assess that TH interventions are equivalent to face-to-face outpatient visits and cost-efficient because of transport savings and unnecessary referrals.

Generally, perceptions of professionals provide a positive view of these tools, and patient satisfaction is noticeable in the majority of the studies. TH allows geodemographic barriers to be overcome and better interprofessional communication and rural and low-income areas are the most benefited locations. Moreover, TH allows for follow-up equivalent to bedside management.

Despite the barriers associated with the adoption and sustainability of TH, some studies reported the effectiveness and cost-effectiveness of TH interventions. Traditionally, professional reluctance had been a significant limitation, but the current COVID-19 pandemic situation has caused TH to become a necessity rather than a choice. Therefore, now is a good time to promote new TH platforms.

## Figures and Tables

**Table 1:** Summary of the studies included in the systematic review.

	Study Type	Patient Intervention	Clinical Results	Cost Evaluation	GRADE
			MANAGEMENT OF ACUTE BURNS		
Ajami et al. (2014) Iran [6]	Systematic review	A review of 30 articles on fast resuscitation and care of burn patients using TH (from 1999 to 2012).	TH proves to be adequate and effective for acute emergency management (rapid resuscitation and patient care), preventing the burn from progressing and becoming infected, and plays a relevant role in improving access to the required experience, increasing professional confidence. It is a useful tool in triage and planning for the treatment of patients.	It can reduce excessive triage for air or ground transportation, saving time and costs. For patients with major burns, TH helped accelerate the preparation of appropriate critical care and justified the costs and risks of air travel. Patients with minor burns were quickly identified for inexpensive ground transportation.	A
Pham et al. (2018) USA [16]	Systematic review	A review of 26 articles analysing burn size estimation (TBSA) (from 1982 to 2017).	Up to 77% of burn victims inappropriately transferred to burn centres from referring hospitals caused by improper use of TBSA. Few studies with limited sample sizes argue that TBSA misestimations significantly affect fluid resuscitation volume; although, the findings suggest that small burns (<20% TBSA) are overestimated and overresuscitated—the opposite of larger burns.	TBSA misestimation is associated with an increased incidence of inappropriate transfers to burn centres and the associated costs. The results suggest that TH could reduce referrals subject to TBSA modification errors, thereby reducing unnecessary costs.	A
Lewis et al. (2012) USA [7]	Systematic review	A review of 31 articles in the emerging field of TH in the management of acute-phase injuries (6 of them related to burn injuries) (from 2004 to 2010).	Focusing on the acute context, mHealth tools provide useful and beneficial plugins for clinical decision-making and support reductions in mortality and morbidity. Especially in rural and resource-poor areas, TH supports better health outcomes and better quality of care.	Although the results of these integrative modalities demonstrated high sensitivity and specificity, high reliability, simplicity, and cost-effectiveness, there are still barriers to the use of TH that limit its wider adoption. The limitations of its adoption include poor infrastructure, limited availability of equipment, and insufficient access to professional training and education.	A
Burgess et al. (2018) Australia [17]	2-group, parallel, single-blinded RCT	“Cool Runnings” app intervention for improving knowledge about risks of hot beverage scalds and of burn first aid in mothers of young children. Participants were women aged 18 years and above with at least one child aged 5–12 months at time of enrolment. In total, 498 participants were recruited via social media and enrolled. At the 6-month follow-up, 244 participants completed the post-test questionnaire.	Intervention group participants achieved significantly greater improvement in overall knowledge post-test than control group participants on both primary outcome measures. These results show that only eight people needed to be exposed to this intervention to improve inadequate overall knowledge to adequate knowledge in one additional person. Participants who remained in the study demonstrated a higher level of education.	Given the low cost and large reach of smartphone apps to deliver content to and engage with targeted populations, smartphone apps can be used for widespread injury prevention campaigns and public health campaigns generally. However, it is important to acknowledge that a change in knowledge does not necessarily reflect a change in behaviour.	B
Wibbenmeyer et al. (2016) USA [18]	Cohort study. Multicentre	Implementation of the addition of video-enhanced TH to the current telephone burn transfer programme in a rural state. Over a 2-year period (2012–2014), 282 patients were enrolled in the study, (59.4% of all burn patients transferred from outside hospitals).	The referring staff was correct in their burn assessment 20% of the time. Video assessment improved the ChargeRN BSA burned and resulted in more accurate fluid resuscitation (*p* = 0.03), changes in both transportation mode (*p* = 0.042), and disposition decisions (*p* = 0.2). The majority of the referring staff found that video-enhanced TH helped them communicate with the burn staff more effectively (3.4 ± 0.37, scale 1–4). This study reports the successful implementation of a video-enhanced TH pilot project in a rural state.	Non-quantitative study. The low cost of the system, coupled with changes in disposition and transportation suggest a significant decrease in healthcare costs associated with the addition of video to a telephone-only transfer programme.	C
Saffle et al. (2009) USA [15]	Cohort study. Multicentre	During the TELE period (from 2005 to 2007), 80 patients were referred, of whom 70 were seen acutely by TH, compared with 28 referrals before instituting TH (PRE-TELE).	Only 31 patients seen by TH received emergency air transport (44.3%), compared with 100% of PRE-TELE patients (*p* < 0.05). TELE patients transported by air had somewhat larger burn sizes (9.0% vs. 6.5% TBSA; *p* = NS) and longer lengths of stay (13.0 days vs. 8.0 days; *p* = NS) than PRE-TELE patients. It demonstrated an improved triage accuracy with face-to-face equivalent TH assessment and proved significantly better than referring clinicians.	TH assessment helped accelerate the provision of appropriate critical care for severely burned patients and justify the expense and risks of air travel. Patients with minor burns were either quickly identified for inexpensive ground transportation or received definitive local care at great cost savings, all without an apparent increase in misclassification.	C
Wallace et al. (2007) UK [19]	Cohort study. Multicentre	During a 12-week prospective study, 11 units with the TH system and 10 units without it regularly made referrals to the Queen Victoria Hospital (QVH). There were 389 referrals from the TH-equipped units and 607 telephone referrals from the non-TH units.	The TH system was used for 246 of the 389 referrals (63%) made from TH-equipped units. It did not document burn size estimation and no clinical outcomes were reported; although, subgroup analysis showed a significantly improved accuracy of triage for minor burns.	No cost savings analysis.	C
Boccara et al. (2017) France [20]	Retrospective study	This retrospective study included 323 patients who were initially assessed by digital images via smartphone, implemented between 2011 and 2016. This procedure only involved patients burned in a small portion of the body surface (i.e., ≤15%), between 15 and 75 years old, and who did not present significant comorbidity.	The initial diagnosis regarding the need for a surgical procedure was accurate in 94.4% (305/323) of the cases. Eleven patients (3.4%) were transferred unnecessarily as they ultimately did not require surgery, and seven patients (2.2%) were ultimately transferred even though the need for surgery was not initially established at the time that the images were viewed. The overall error rate was 5.6% (18/323). This could equally be a result of burn progression rather than incorrect evaluation of the initial image. The delay in treatment did not adversely affect life-threatening, functional, or aesthetic prognoses.	Out of the 222 patients not hospitalised on an emergency basis, only seven ultimately underwent surgery for a straightforward excision–skin graft. This low error rate (3.2%) and the lack of observed injury supported the development of photograph-based opinions. The cost savings and the freeing-up of the burn centre resources were very substantial during this period, thanks to this TH system.	D
Saffle et al. (2004) USA [21]	Retrospective study	In total, 225 acutely burned patients from 2000 to 2001 who were transported to the facility by air from referring hospitals in nine states. They wanted to study whether TH evaluation before transport could have significantly altered initial treatment decisions.	Out of the 225 patients, only 60% were classified as air-transport appropriate. The mean burn size calculated by burn centre physicians was 19.7% TBSA, whereas that calculated by referring physicians was 29%. In 92 cases, over- or underestimation of burn size by referring physicians was as much as 560%. TH evaluation before transport might have significantly altered transport decisions or care.	Some patients obviously met more than one of these criteria. The TH group had shorter lengths of hospital stay than the other patients (13.5 ± 2 days vs. 24.2 ± 3 days, *p* < 0.05) and, correspondingly, lower hospital charges. Air transport charges exceeded hospital charges in 21 cases.	D
Mohr et al. (2017) USA [22]	Cohort study	In total, 2837 patients were treated to describe patient-level factors associated with TH consultation in an emergency department (ED) and to measure the association between TH consultation and interhospital transfer from 2008 to 2014.	No differences were observed in clinical outcomes. TH was consulted for 11% of all trauma patients in TH-capable EDs. Factors associated with TH consultation included a higher Injury Severity Score. Adjusting for the severity of illness, injury mechanism, and type of injury, TH use was not associated with interhospital transfer (adjusted odds ratio = 1.28, 95% confidence interval = 0.94 to 1.75).	No cost analysis.	C
Wallace et al. (2008) UK [8]	Cohort study	Study conducted in different phases. (1) Ten-week retrospective evaluation of the TH system with 973 referrals from 53 different sites. (2) Twelve-week prospective cohort study to investigate changes in patient management from TH-assisted referrals compared to telephone-only referrals. In total, 996 referrals were received from over 60 different sites.	(1) In total, 452 patients were referred from the sites with TH, and TH was used for 42% of these patients. Referring clinicians were pleased with the TH system, finding it easy to use and helpful in the referral process. They also expressed an improvement in the clarity of information. (2) Of 389 referrals, the TH system was used for 243 (63%). A significant difference was noted in the management of patients with and without the availability of TH. Significantly fewer patients needed to come for further assessment and more patients could be directly booked for definitive care in a Day Surgery Unit (10.5%). A decrease in the number of occasions when the QVH was unable to accept a referral due to a lack of capacity was observed compared to telephone advice only.	The authors found no evidence of cost savings for the QVH trust and only anecdotal patient cost savings. The capital outlay was significant (£70,000) for the installation of the computer network lines, equipment, and software.	C
Hop et al. (2014) The Netherlands [23]	Cohort study	This study examined the reliability and validity of using 50 randomly selected photographs taken on day 0–1 post-burn by seven burn experts and eight referring physicians to assess both burn size and depth from one general hospital.	Experts (ICCs of 0.83 and 0.87), but not referring physicians (ICCs of 0.68 and 0.78), could assess burn size from photographs both reliably and validly. Neither experts (0.38 and 0.28) nor referring physicians (0.24 and 0.13) could assess burn depth either reliably or validly, or the indication for surgery. The agreement between assessors regarding referral indication was low.	No cost analysis. Future research should also address the actual impact and cost-effectiveness of the introduction of TH, e.g., the prevention of unnecessary referrals, in a clinical trial.	C
Cai et al. (2016) Nepal/USA [12]	Cohort study	A prospective study conducted with 17 individuals with healed burn scars in Nepal. Three independent observers (one physically present and two remote observers in the United States) assessed 85 burn scars to test the reliability of the Patient and Observer Scar Assessment Scale (POSAS) using live videoconferencing.	The single-rater reliability of the POSAS was acceptable (ICC > 0.70) in overall opinion, thickness, pliability, and surface area. The average rater reliability for three observers was acceptable (ICC > 0.70) for all parameters except for vascularity. When comparing patients’ and observers’ overall opinion scores, the patients’ scores were consistently worse.	No cost analysis. This study demonstrated that an off-the-shelf camera smartphone is sufficient to transmit a reliable video-feed of burn scars from Nepal to the US. Videoconferencing offers an acceptable low-cost solution applicable to most resource-limited healthcare environments.	D
Shokrollahi et al. (2007) UK [24]	Case-control	An investigation into the accuracy of assessment of TBSA and depth in 31 patients with minor burns using a basic camera-equipped mobile phone, assessed at the Welsh Centre for Burns and Plastic Surgery.	It demonstrated a good correlation of burn size (correlation coefficient r = 0.91) estimation, though only for small burn sizes (<5%TBSA (mean 1.2%)). Using the images, assessors could reliably differentiate full-thickness burns from partial-thickness burns in almost all cases (94%, n = 29).	No cost analysis. Within minutes, an emergency department was able to transmit accurate images to the centre, enabling good decisions to be made related to the appropriateness of transfer, interim dressings, and fluid resuscitation, with profound implications for the quality of patient care as well as cost.	D
Parvizi et al. (2014) Austria [25]	Cross-sectional study	At two international burn meetings, a survey containing three pictures of burn patients was conducted. Eighty specialists were asked to give a burn extent estimation. The same burn pictures were transferred to a computer system and the TBSA in % was calculated by the BurnCase 3D software and the estimations were compared.	The majority of respondents were specialists (32), residents (27), and nursing staff (21). The preferred methods for burn extent estimation were the Rule of Nines (38%), the Rule of Palm (37%), and the Lund-Browder chart (18%). The analysis showed very high deviations of TBSA across the participants, even in the group of experts. In comparison to a computer-aided method, the authors found a massive overestimation of up to 230%.	No cost analysis. BurnCase 3D brought an objective extent estimation that could have a true impact on the quality of treatment in burns. In acute burn care, TH had great potential to help guide decisions regarding triage and transfer based on TBSA, burn depth, patient age, and injury mechanism.	D
Kiser et al. (2013) Malawi/USA [26]	Cohort study	In the burn unit at Kamuzu Central Hospital (KCH), Malawi, 39 burn patients (50 wounds) were clinically assisted and also photographed by an experienced clinician over a 2-month period in 2011. Then, these photographs were reviewed by two blinded burn clinicians after 4–6 weeks. The correlation between clinical assessment and photographic evaluation was calculated using the Kappa score and Pearson’s correlation coefficient.	Pearson’s correlation coefficients for TBSA agreement between clinical examination and photograph review by experts 1 and 2 were 0.96 and 0.93 (*p* < 0.001), respectively. Pearson’s correlation coefficients comparing experts 1 and 2 to the gold standard were the proportion of full-thickness burn (0.88 and 0.81, *p* < 0.001), and epithelialised superficial burn (0.89 and 0.55, *p* < 0.001). Kappa scores were significant for wound evolution (0.57 and 0.64, *p* < 0.001), and prognosis (0.80 and 0.80, *p* < 0.001).	No cost analysis. Burn assessment with digital photography was a valid and affordable alternative to direct clinical examination, alleviating access issues to burn care in developing countries.	C
Smith et al. (2007) Australia [27]	Case-control	Over a 5-year period, a novel telepaediatric service (videoconferencing) was set up for selected regional hospitals in Queensland. In total, 1499 consultations were conducted for a broad range of paediatric subspecialities including burns.	No clinical results	Total cost of providing 1499 consultations was AUD 955,996, but the estimated cost without this service at the hospital was AUD 1,553,264; thus, telepaediatric services resulted in a net saving of approximately AUD 600,000 to the health service provider. TM was cheaper than conventional outpatient service after 774 consultations. The state reimburses the patients’ travel expenses.	D
Gacto-Sánchez et al. (2020) Spain [28]	Cross-sectional study	Diagnostic test validation study made by TH (through an App designed for this project) to 202 patients with acute burns between July 1 and 23 October 2018.	All images were valid for diagnosis. Quality evaluation was “very good” (52%) and good (43%). The intra-observer concordance was k = 0.94 (95% CI: 0.90 0.97). Interobserver concordance: k = 0.95 (95% CI: 0.910.99). The results highlight a very high sensitivity (99.40%) and specificity (100%).	The TH detected that 83.17% of the patients attending the BU could have been managed on as outpatient basis. 49.44 min for tele-response report (95% CI 45.89-52.67; range 2-138) vs. 243.60 min to assist the patient at the BU (95% CI 224.05-266.75; range 19-1620). The study optimizes the use of resources (urgency consultations, medicalized transportation) (non-data presented).	D
Basaran et al. (2020) Turkey [29]	Cross-sectional study	Study of reliability of TH assessment of burn patients and preference of patients to use TH.	TH examination resulted in an agreement in terms of burn depth, decision of hospitalisation, and a high concordance for TBSA evaluation between face-to-face examination and TH group.	Although WhatsApp is a reliable method, the majority of patients preferred a face-to-face follow-up.	D
Garber et al. (2020) USA [30]	Cross-sectional study	155 burn patients from rural areas were referred by telephone to provide initial triage and the need or not to transfer to the reference hospital, compared to the decision based solely on the telephone conversation.	In total, 24.5% of patient images changed the initial decision transfer, and 75.5% confirmed the initial care plan. Of the cases that required a change of plan, 60.5% were they went down to outpatient care, and 39.5% went up to transfer.	They saved between USD 150,000 to USD 180,000 in air freight costs alone. The authors do not calculate the additional cost of hospitalisation that the patient would have incurred.	D
Carmichael-et al. (2020) USA [31]	Retrospective study	To assess the efficacy of a mobile app in the triage decisions of burn patients.	The Burn mobile app can be used to improve triage decisions in patients with intermediate-size burns measuring 1–10% TBSA. The Burn mobile app can be used in a HIPPA-compliant manner.	The cost savings to the system and patients were estimated to be nearly USD 100 per patient.	D
		FOLLOW-UP OF BURN PATIENTS			
Liu et al. (2017) USA [32]	Retrospective study	A retrospective review was performed on 29 patients enrolled in 73 virtual visits through the TH/rehabilitation programme between 2013 and 2014.	Videoconferencing between a burn centre and rehabilitation hospital streamlined patient care and reduced healthcare costs, while maintaining quality of care and patient satisfaction.	Total savings of USD 101,110 in transportation costs were achieved by eliminating 146 ambulance transfers. The reduced time of virtual visits resulted in savings of 6.8 days in outpatient management. Early discharge was facilitated, and savings of 80 bed days were estimated. The rehabilitation hospital saved an average of 2.5 days by eliminating travel. No unplanned readmissions from the rehabilitation hospital to the burn hospital were observed during the study. The patient satisfaction surveys showed 100% satisfaction, especially related to the trips saved. The rehabilitation centre had reduced interruptions, thereby improving its efficiency.	D
Hickey et al. (2017) USA [33]	Retrospective study	A review of 31 burn patients participating in Interactive Home Telehealth (IHT) visits for follow-up burn care using videoconferencing over the course of 15 months (from 2015 to 2016).	There were no unplanned readmissions and no complications. Of 31 total patients, burn surgeons treated 26, physiatrists treated 4, and psychiatrists treated 6, with a mean length of time for the IHT consultation of 10.8 min, 17.2 min, and 30 min, respectively. Of the 34 encounters with burn surgeons, 23 (67.7%) were supplemented with high-resolution images.	The average roundtrip travel distance saved was 188 miles (range 4–822 miles). The average roundtrip travel time saved was 201 min (range 20–564 min). Five connectivity issues were reported, none of which prevented the completion of the visit.	D
Garcia et al. (2018) USA [34]	Case-control	The authors retrospectively reviewed clinical outcomes and usability in paediatric partial thickness burn patients treated using the TeleBurn App (32) through text and image messaging, videoconferencing, and instructional videos, compared to standard therapy alone (35), between 2016 and 2017.	Most of the patients (74%) who were offered the app used it as their primary source of follow-up care. This group had no wound infections or unexpected returns to a clinic or hospital. Both the TeleBurn App and standard therapy groups had similar burn severity, age, and burn mechanism. Mean healing time was shorter in the app group (11.6 ± 4.7 days) vs. standard therapy (14.3 ± 5.4 (*p* = 0.03)) with fewer clinical encounters (0.93 ± 0.6) vs. standard therapy (3.3 ± 1.0 (*p* = 0.001)). Adherence to completion of therapy in patients using the app was 80% vs. 64% with standard therapy.	No cost analysis. While understudied, the cost of distributing and licencing an app-based care model would likely be significantly lower than the cost of former TH hub-and-spoke models used in burn care.	D
Nguyen et al. (2004) USA [35]	Cohort study	Evaluation of 1000 burn follow-up visits with 294 patients via TH over a 5-year period to identify the barriers and benefits specific to burn care. Travel costs and financial data were evaluated.	No clinical outcome analysed. Psychology, therapy, and surgical care were delivered during the virtual visits. Subjective improvement in local liaison and quality care. The benefits of TH included a decrease in travel, improved continuity of care, and increased access to specialised consultants.	Total costs for the 1000 TH follow-up visits were USD 145,522, averaging USD 146 per visit. TH burn visits were a cost-effective clinical alternative for the patient. In contrast, TH could be a financial burden to healthcare systems and inefficient for healthcare providers.	C
Redlick et al. (2002) Canada [36]	Cohort study	This study evaluated (1) patient and (2) physician satisfaction with 14 teleconsultations (video/audio communication) in follow-up burn care and assessed the costs and benefits of these teleconsultations in 1999.	(1) In total, 67% of patients felt that talking to the specialist and asking the specialist questions in their teleconsultation was much easier than a face-to-face visit, whereas 33% of patients found that teleconsultations were equal to an in-person visit. The patients indicated that they were very satisfied with their appointments and that the teleconsultations were much better than traveling to out-of-town specialists. (2) The consulting physician reported that patients presented with the same types of problems as those seen in regular practice. The consulting physician was also very satisfied because they saved the patients and burn care team time and money and allowed the burn care team to plan surgeries and rehabilitation strategies more efficiently. The only reported difficulty was with discussing personal issues with patients during teleconsultations.	(1) On average, the patients’ teleconsultations were completed in only 2.7 h. This time investment was significantly shorter than the estimated trip of more than two days to the burn unit (*p* < 0.0001). The average expense per patient was CAD 16.66 for a teleconsultation, which was significantly less than the estimated average cost per patient of CAD 615.74 for an out-of-town consultation (*p* < 0.01). (2) The average time was 19.5 min. The average cost of a teleconsultation was CAD 57.09 in specialist fees and CAD 11.12 in telecommunication fees (19.5 min at CAD 0.57 per minute).	C
Smith et al. (2004) Australia [37]	Cohort study	This study compared the use of videoconferencing for the assessment of burns with conventional, face-to-face (FTF) assessments. A total of 35 children with a previous burn injury were studied.	This study confirms that the quality of information collected during a videoconference appointment is comparable to that collected during a traditional, FTF appointment for a follow-up burns consultation.	No cost analysis.	C
		ACUTE BURN MANAGEMENT AND FOLLOW-UP			
Wallace et al. (2012) UK [2]	Systematic review	A review of the findings of 24 articles in burn care to assess the evidence for the use of TH in acute burn care and outpatient treatment (from 1993 to 2010). Mostly case series studies.	The studies suggested that TH in the management of acute burns was feasible (TBSA evaluation, emergency triage, and need for interventions) and might be as good as face-to-face evaluation, aiding clinical decision-making. However, comparative studies showing TH superiority or equality were lacking. Further education was needed to familiarise professionals with technology.	Dependent on the country (healthcare system and burn infrastructure) and who pays. Greater patient convenience and substantially fewer costs and less time (more satisfied). The initial cost was higher for the medical care provider.	A
Vyas et al. (2017) USA [38]	Systematic review	A review of the findings of 23 articles about the use of TH in plastic and reconstructive surgery, and dermatology, five of which were on burn management (from 2010 to 2017).	All 23 articles reported TH benefits, which frequently related to better post-operative monitoring, greater access to rural settings without affecting the quality of care provided, and cost savings. TH improved the coordination of care and management of burn wounds, facilitated interprofessional collaboration over time and space, and saved a significant number of unnecessary referrals. Greater commitment, standardisation, and regulation were required. Privacy and security remained unresolved concerns.	Although the studies suggested that TH could produce cost savings and better results, larger and more general studies were needed.	A
Hoseini et al. (2016) Iran [39]	Systematic review	A review of 32 articles about TH applications in the treatment of burn patients (from 2000 to March 2016).	TH could help reduce possible errors in the categorisation of the burned patient. Remote monitoring had been shown to be successful in patients residing in distant areas. There was still resistance from doctors as well as legal challenges. Teleconsultation also led to success for outpatient injuries, though the potential for committing decision-making errors should not be underestimated. Numerous studies showed that the results of the burn size estimation and evaluation via imaging and TH were very close to the results from face-to-face evaluation and diagnosis.	The conventional technique showed a considerable percentage of unnecessary transfers. According to several findings, the use of TH to assess burns resulted in savings of money and time, increased productivity, reduced referrals, and unnecessary transportation, with the correct and most efficient method for patient transfer and treatment being chosen.	A
Wiktor et al. (2018) USA [40]	Retrospective multicentre study	A retrospective review was conducted of referrals from 2016 to 2017 at three regional burn centres utilising the Burn App to facilitate triage of patients by allowing referring providers to send encrypted photos, thus enhancing the telephone consultation process.	A total of 2011 consults were placed using the mobile phone app from 294 different referring facilities spanning seven states. Overall Burn App utilisation among enrolled referring centres was 45% (range 39–48%). Most patients were referred to outpatient clinics for continued burn care (59%), 22% were admitted, and 18% received care at local facilities. The application seemed to be a useful tool for patient triage.	As telehealth and technology were more readily utilised, the question of whether or not a platform such as the Burn App improved triage decisions, affected patient care, and ultimately reduced costs still required further study.	D
Martínez et al. (2018) South Africa [41]	Retrospective study	A review was conducted of all consultations using WhatsApp over an 18-month period, received by the burn centre’s two senior medical practitioners from 2015 to 2016.	838 communications and 1562 different clinical consultations were described; 486 (58%) intrahospital and 352 (42%) between centres. Most of the images received were of adequate quality for the evaluation of depth, focus, colour, and clinical relevance. The use of WhatsApp in daily burn care processes significantly improved the quality of paediatric referrals to specialised burn services. Unnecessary referrals and outpatient visits were reduced, continuing medical education was facilitated, and care for large burns was improved through more effective communication.	Outpatient visits were significantly reduced during the study period. It was estimated that up to 160 unnecessary admissions were also avoided as a result of better triage that translated into considerable cost savings for the institution and better distribution of resources.	D
Russell et al. (2015) USA [42]	Retrospective study	This retrospective review from 2005 to 2014 evaluated burn TH visits and financial reimbursement during (2005–2007) and after (2008–2014) a Technology Opportunities Program (TOP) grant to a regional burn centre.	In 2005, it had 12 TH visits, which increased to 458 in 2014. It was possible to demonstrate that evaluation of burn extent and depth via video was essentially equivalent to face-to-face examination. Patient and provider satisfaction was extremely high. During the 26 months that the TOP grant was active, consultations at the burn centre almost tripled, air transports decreased from 100% to 44% of consults, and burn severity of those patients transported increased.	Over 3 years, they admitted 42 patients after initial TH evaluation that generated more than USD 4 million in hospital revenue. A separate sampling of 24% of teleburn visits from 2010 to 2011 showed that these visits directly resulted in USD 4.2 million in revenue to the University of Utah. This success has resulted in expanded institutional efforts in TH. Furthermore, because of the profitability of the teleburn enterprise, the hospital has assumed responsibility for operating costs, including equipment upgrades, and now budgets these as operating costs for the TH programme.	D
Turk et al. (2011) Turkey [43]	Cohort study	This study investigated the use of TH in decision-making and follow-up of 187 burn patients, in 2003 and up to 2009, all of whom had teleconsultations (audiovisual) with the same burn surgeon at the Ankara Burn Referral Centre.	Over a 66-month period, 525 televisits with 187 patients were carried out. As a result, 21 patients (11.2%) were transferred to the referral centre in Ankara. The mean TBSA was 23.3 ± 17.8%. The mean hospital stay was 16.4 ± 13.4 days. In total, 157 patients were discharged after successful burn therapy (84%). Nine (4.8%) died owing to multiorgan failure and sepsis. The number of deceased patients, televisits, and transferred patients decreased over time.	No cost analysis. However, TH was said to be appropriate and cost-effective for the treatment and follow-up of patients in burn units by personnel with limited experience. The potential benefits of TH included reductions in patient travel costs, continuity of care, and access to specialised health consultations in remote areas.	C
McWilliams et al. (2016) Australia [44]	Retrospective audit study	From 2005 to 2013, 904 patients were referred to the paediatric Burns Telehealth Service in Western Australia. This is a retrospective chart audit of avoided transfers and bed days, and the avoided associated costs to the tertiary burn unit and patient travel funding.	No clinical outcomes.	Over an 8-year period, the audit identified 4905 avoided inpatient bed days, 1763 avoided follow-up review transfers, and 364 avoided acute patient transfers for a total of 1312 paediatric burn patients because of this telehealth service. The paper presented the derivation of these outcomes and an estimation of their cost savings in 2012–2013 of AUD 1.89 million.	D
Head et al. (2022) USA [45]	Cohort study	To assess the efficacy and cost savings of virtual visits for acute, outpatient burn care in the home setting during a 6-month timeframe of the COVID-19 pandemic.	There were no significant differences in burn % TBSA, depth, cause, number of unplanned readmissions, number of unplanned reoperations, or complications.	Virtual visits offer significant cost savings for the patient and can be as effective as traditional face-to-face visits in the outpatient burn care setting.	C
Smith et al. (2007) Australia [46]	Retrospective study	Review of the first 1000 burns consultations conducted by the telepaediatric service over a 6-year timeframe.	No clinical outcomes.	Assuming that each consultation required a return journey, and the paediatric patients were accompanied by a parent or carer, the total distance saved would be over 1.4 million km.	D

GRADE: The Grading of Recommendations, Assessment, Development, and Evaluation approach. LOS: Length of hospital stay; TBSA: Total body surface area; TH: Telehealth.

**Table 2:** Type of studies included in the systematic review.

Study Type	Number of Articles Selected
Systematic review	6
Randomised control trial	1
Cohort study	13
Retrospective study	10
Cross-sectional study	4
Case-control study	3
TOTAL	37 articles

## Data Availability

Not applicable.

## Supplementary Materials

The following supporting information can be downloaded at: https://www.mdpi.com/article/10.3390/ijerph20043161/s1, Figure S1: Number of articles published per year.
