# Supplementary figures and images for: Utility of Telehealth Platforms Applied to Burns Management: A Systematic Review
Source: Int J Environ Res Public Health. 2023 Feb 10;20(4):3161. doi: 10.3390/ijerph20043161 (PMC9968161; doi:10.3390/ijerph20043161)

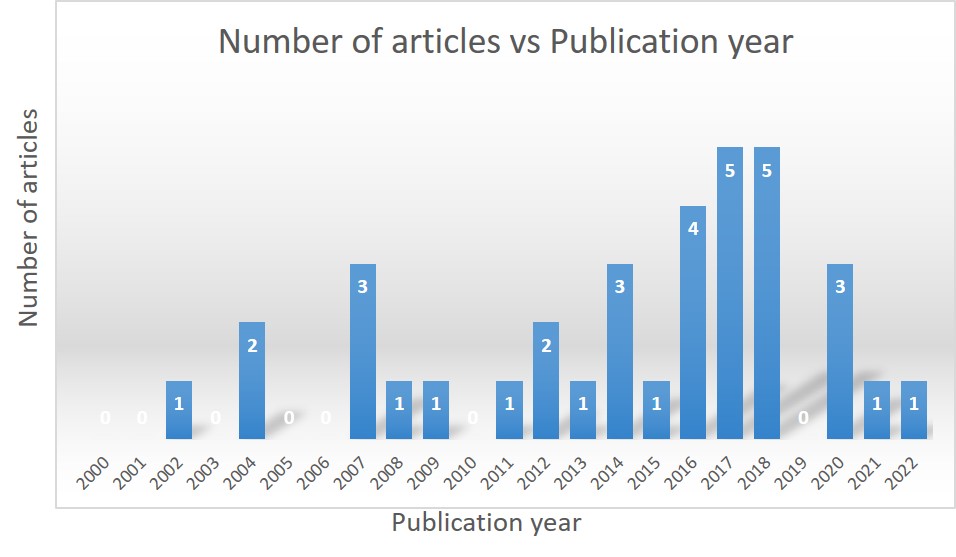

Supplement: Supplementary file 1 [file ijerph-20-03161-s001.zip › ijerph-2130680-supplementary.jpg]
